# Higher than expected incident cases of spinal bulbar muscular atrophy in western Canada

**DOI:** 10.1093/brain/awae052

**Published:** 2024-02-14

**Authors:** Ryan Lamont, Malcolm King, Alexandra King, Kerri Schellenberg, Gerald Pfeffer

**Affiliations:** Department of Medical Genetics, University of Calgary, Calgary, AB, T2N 4N1, Canada; College of Medicine, University of Saskatchewan, Saskatoon, SK, S7N 5E5, Canada; Mississaugas of the Credit First Nation, ON, Canada; College of Medicine, University of Saskatchewan, Saskatoon, SK, S7N 5E5, Canada; Nipissing First Nation, ON, Canada; College of Medicine, University of Saskatchewan, Saskatoon, SK, S7N 5E5, Canada; Department of Medical Genetics, University of Calgary, Calgary, AB, T2N 4N1, Canada; Hotchkiss Brain Institute, Cumming School of Medicine, Department of Clinical Neurosciences, University of Calgary, Calgary, AN, T2N 4N1, Canada

Spinal bulbar muscular atrophy (also known as Kennedy disease) was recently described to have a high prevalence among Indigenous people in the prairie provinces of western Canada due to a genetic founder effect.^1^ In this work, the prevalence was estimated at 1/6802 (i.e: 14.7/100 000), the highest known prevalence in the world, but based on the study design was considered to be an underestimate. This compares to estimates of the global prevalence at 1–2/100 000. Recently, an analysis of whole genome sequence data from large genomic datasets suggests a much higher-than-expected carrier frequency of the *AR* expansion, with a possible disease prevalence as high as 1/6887 males (or approximately 1/13 774 population).^2^ The massive underdiagnosis of spinal bulbar muscular atrophy is likely to be multifactorial and may be related to variable penetrance, expressivity and social determinants of health, among other factors.

To obtain further evidence regarding apparent underdiagnosis of spinal bulbar muscular atrophy, we conducted a survey of diagnostic testing results for the *AR* trinucleotide repeat at the Molecular Genetics Laboratory at the Alberta Children’s Hospital. Since 2018, this laboratory has received and reported on all diagnostic testing for spinal bulbar muscular atrophy for the provinces of Alberta and Saskatchewan, and the Northwest Territories, covering a total population of 5.59 million people. Research ethics board approval was obtained from the University of Calgary Conjoint Health Research Ethics Board (REB23-0215). Community consent was obtained after consultation with our patient advisory council, an Indigenous Community Guiding Circle consisting of five people affected by spinal bulbar muscular atrophy from Indigenous communities, and a Saulteaux Knowledge Holder from an affected community (the Key First Nation), and specific agreement for the submission and publication of this letter was obtained during a meeting on 8 November 2023.

We performed a retrospective chart review of diagnostic and carrier testing, reporting all cases with *AR* exon 1 expansions containing 38 or more CAG repeats. Clinical information collected from the records included sex and province of residence. Prevalence of incident cases was calculated relative to the total population of each region (Statistics Canada). During the 5.5-year period (January 2018 until July 2023) we identified 59 new cases of spinal bulbar muscular atrophy. The incident cases over this period are summarized in Table 1 and is 1.06/100 000 population across the three regions covered by our laboratory.

**Table 1:** Incident cases of spinal bulbar muscular atrophy over the 5.5-year period

Region	Regional population in 100 000	Positive male cases, *n*	Positive female carrier cases, *n*	Total positive cases	Incident cases/100 000^a^
Alberta	43.71	39	8	47	0.89
Saskatchewan	11.74	19	1	20	1.62
Northwest Territories	0.448	1	0	1	2.23
Total	55.9	59	9	68	1.06

^a^Male spinal bulbar muscular atrophy cases per total population (males + females).

The incident cases from only 5.5 years’ worth of testing in the regions covered by our diagnostic laboratory is similar to the estimated total global prevalence. This certainly suggests that the total prevalence in our regions is likely to be several-fold higher. This only includes cases which have been clinically ascertained, and the true prevalence is likely underestimated due to diagnostic delay, late onset of symptoms, or lack of access to testing and healthcare resources. There is also an important interaction of Indigenous health determinants that affects the ascertainment of cases.

It is noteworthy that a very small number of female carriers were identified by our laboratory during this period. This indicates that family genetic counselling and carrier testing may be under-utilized in our regions. Better access to these resources would be of benefit to help people obtain education about risks of passing on the mutation and to improve diagnosis in family members.

We have previously reported the high prevalence of spinal bulbar muscular atrophy among Indigenous peoples in these regions. Our experience is that over 80% of patients in our clinics self-report Indigenous ancestry, usually from Cree, Saulteaux or Métis Nations. If it holds true that 80% of these positive results are from Indigenous people, then when compared to the Indigenous population of these regions (approximately 491 000), this would also indicate a very high number of incident cases over the 5.5 year period (11/100 000). For comparison, we had previously estimated the prevalence in this population as 14.7/100 000, and these newer data from incident cases suggest the true total prevalence is likely to be several-fold higher. Given the findings of Zanovello and colleagues,^2^ the carrier frequency of the *AR* expansion is likely also underestimated by at least 10-fold, which explains findings such as the presence of homozygous females with the *AR* expansion from an Indigenous community in Manitoba,^3^ an adjacent province. We emphasize the importance of ongoing engagement with communities to aid in the ascertainment of cases. This will help affected patients gain access to necessary supportive care resources, and importantly, access to future therapies that are currently in development for spinal bulbar muscular atrophy.^4^

## Data Availability

The authors confirm that the data supporting the findings of this study are available within the article.

## References

[1] Leckie JN , Joel MM, Martens K, et al Highly elevated prevalence of spinobulbar muscular atrophy in indigenous communities in Canada due to a founder effect. Neurol Genet. 2021;7:e607. 34250227 10.1212/NXG.0000000000000607 PMC8267784

[2] Zanovello M , Ibáñez K, Brown AL, et al Unexpected frequency of the pathogenic AR CAG repeat expansion in the general population. Brain. 2023;146:2723–2729. 36797998 10.1093/brain/awad050 PMC10316764

[3] Schmidt BJ , Greenberg CR, Allingham-Hawkins DJ, Spriggs E. Expression of X-linked bulbospinal muscular atrophy (Kennedy disease) in two homozygous women. Neurology. 2002;59:770–772. 12221177 10.1212/wnl.59.5.770

[4] Wilton-Clark H , Al-Aghbari A, Yang J, Yokota T. Advancing epidemiology and genetic approaches for the treatment of spinal and bulbar muscular atrophy: Focus on prevalence in the indigenous population of Western Canada. Genes (Basel). 2023;14:1634. 37628685 10.3390/genes14081634 PMC10454234

